# A Case of Advanced Non-Small-Cell Lung Cancer With Response to Alectinib and Favorable Quality of Life

**DOI:** 10.7759/cureus.21170

**Published:** 2022-01-12

**Authors:** Dhan B Shrestha, Vijay Ketan Reddy, Suman Gaire, Mohammed Kassem

**Affiliations:** 1 Department of Internal Medicine, Mount Sinai Hospital, Chicago, USA; 2 Department of Emergency Medicine, Palpa Hospital, Palpa, NPL; 3 Department of Hematology and Oncology, Mount Sinai Hospital, Chicago, USA

**Keywords:** advanced non-small-cell lung cancer, adenocarcinoma, alk inhibitors, alectinib, alk-positive, nsclc

## Abstract

Lung cancer is the leading cause of cancer death globally and in the United States. Non-small-cell lung cancer (NSCLC) accounts for approximately 85% of lung cancer cases. A progressive increase in morbidity and mortality is seen with advanced disease. Identifying specific driver mutations, such as anaplastic lymphoma kinase (ALK) mutations and directed therapy, has improved the quality of life and survival in ALK-positive NSCLC patients. Here, we present the case of a 37-year-old female who was diagnosed with stage IV NSCLC (adenocarcinoma) with a positive ALK mutation six years ago. Our case report highlights a rare ALK mutation NSCLC treated with targeted ALK inhibitor therapy. Despite having advanced-stage cancer, the treatment significantly impacted her survival with an improved quality of life.

## Introduction

Lung cancer is the second most common malignancy, with 2,206,771 new cases reported in 2020. It is also the leading cause of cancer deaths, with 1,796,144 cases reported globally [1]. In the United States, lung cancer is the leading cause of cancer-related deaths in males and females and is the third most common malignancy by annual incidence [2]. Non-small-cell lung cancer (NSCLC) accounts for approximately 85% of lung cancer cases [3]. The overall incidence of lung cancer is higher in males than in females [4]. However, in individuals less than 50 years of age, the female incidence has been higher than the male incidence in the United States [5]. Furthermore, the incidence of lung cancer increases with age, with the highest incidence seen in the 80-84-year age group. The incidence is particularly low in individuals less than 40 years of age, with an incidence of around 5/1,000,000 [4].

The treatment for advanced NSCLC has been influenced by the presence or absence of driver mutations. One of these mutations is anaplastic lymphoma kinase (ALK) rearrangements. ALK belongs to a group of transmembrane receptor kinases, and its aberration is associated with various cancers. ALK rearrangement is present in about 5% of NSCLC cases [6]. The first ALK fusion gene was found in 2007 in NSCLC. A slight inversion in chromosome 2p had resulted in echinoderm microtubule-associated protein-like 4 (EML4) and the ALK fusion gene [7]. The EML4 ALK fusion gene is more likely to be present in light or non-smokers and is associated with adenocarcinoma histology [8]. The ALK gene rearrangements or the resulting fusion proteins can be detected in tumor or plasma specimens by next-generation sequencing (NGS), fluorescent in-situ hybridization (FISH), or immunohistochemistry (IHC).

ALK-positive NSCLC survival has increased on treatment with a class of ALK drugs inhibitors compared to conventional chemotherapy. Five ALK inhibitors have been approved by the US Food and Drug Administration for ALK-positive NSCLC, namely, crizotinib, ceritinib, alectinib, brigatinib, and lorlatinib.

## Case presentation

**Figure 1:**
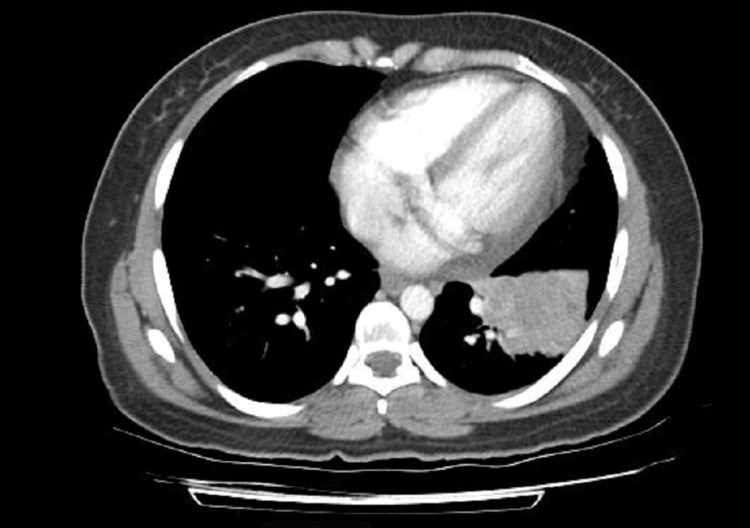
Lobulated mass in the lower lobe of the lung.

A 37-year-old female presented six years back with painless, progressive left axillary, cervical, and supraclavicular lymphadenopathy for two months. She had a non-productive cough for the same duration. She was a former, occasional smoker, a social drinker, and did not have a history of substance abuse. No significant family history of malignancy was reported. Fine needle aspiration followed by a core axillary lymph node biopsy was performed. The histopathology confirmed her diagnosis of metastatic adenocarcinoma, with ALK-positive and epidermal growth factor mutation-negative status. A computed tomography (CT) scan of the chest, abdomen, and pelvis revealed a large, lobulated mass in the left lower lobe of the lung, measuring 5.5 × 4.4 cm, with narrowing of the left lobar bronchus (Figure 1).

**Figure 2:**
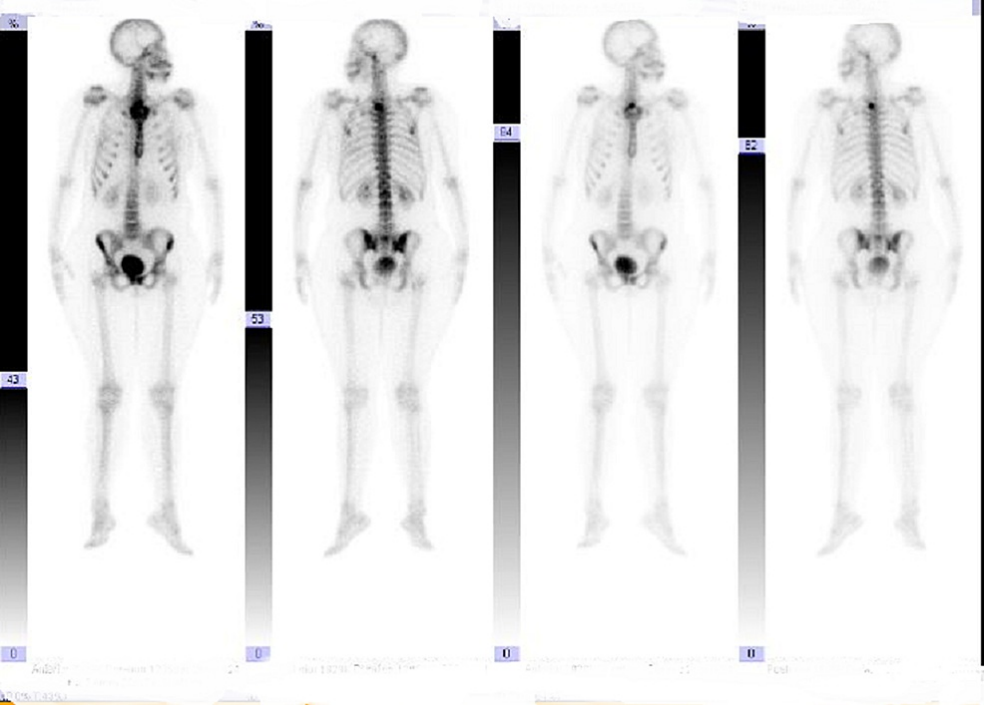
Bone scan suggestive of T2 vertebral body metastasis.

Furthermore, a hypodense lesion was visualized on the undersurface of the T2 vertebral body. There were multiple enlarged mediastinal lymph nodes. A CT scan of the neck, with contrast, revealed enlarged left pectoral and left sub-clavicular lymph nodes. A magnetic resonance imaging (MRI) of the brain was negative for metastasis. A bone scan revealed a suspicious osteoblastic metastasis in the T2 vertebral body (Figure 2). An MRI of the cervical and thoracic spine showed a T1 hypointense lesion in the second thoracic (T2) vertebral body, suggesting metastasis.

The patient was initially started on combination chemotherapy with carboplatin, paclitaxel, and bevacizumab until her ALK status was determined. ALK status determination can usually take up to four weeks from a tissue biopsy. She was then commenced on crizotinib 250 mg twice daily. A very good response to crizotinib was noted within five months of therapy initiation. The T2 vertebral lesion revealed sclerosis, and the left lower lobe mass decreased to a size of 2.2 × 2.1 × 2.1 cm. A zoledronic acid, calcium, and vitamin D regimen was also started. Following crizotinib initiation, she complained of visual symptoms, such as floaters, flashes of light, and photopsia. She was diagnosed with scintillating scotomas, which are now well recognized adverse effects of crizotinib.

Eighteen months after initiation of crizotinib, a progression was noted, with the left lower lobe mass measuring 2.7 × 2.4 × 3.2 cm. An MRI of the brain was negative for any metastatic lesions. Crizotinib was then changed to alectinib 150 mg four tablets twice daily.

**Figure 3:**
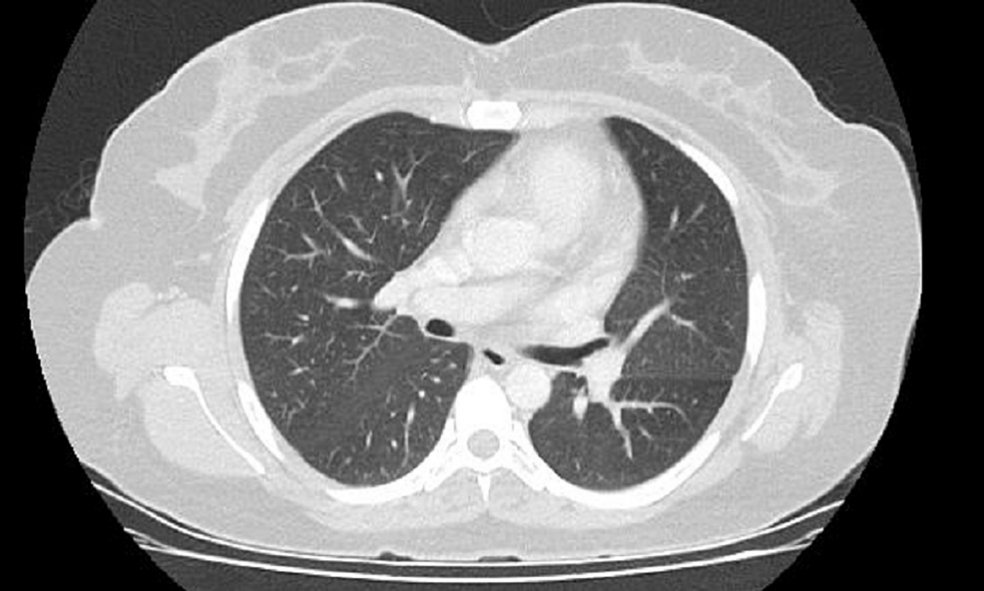
Recent CT scan of the chest showing response to alectinib. CT: computed tomography

After initiating alectinib, the patient reported adverse effects, such as constipation, episodes of dizziness, and visual spots. The constipation was treated with laxatives. She has continued to have an excellent response to alectinib, documented with the gradual decrease in the size of the lung lesion (Figure 3) and no apparent, metabolically active disease according to positron emission tomography-CT scans. She has had an Eastern Cooperative Oncology Group Performance Status score of zero with an excellent quality of life.

## Discussion

We have presented a case of ALK-positive, stage IV, advanced NSCLC. The first-line treatment has been driven by the presence of a driver mutation in patients with advanced NSCLC. To avoid delays in initiating therapy in patients with metastatic cancer, patients are usually started on chemotherapy and subsequently changed to targeted drug therapy if a driver mutation is identified. Such was the case with our patient. In our case, zoledronic acid was also given for osteoclast inhibition to prevent the skeletal complication of metastasis.

All patients with advanced adenocarcinoma should be tested for the ALK fusion gene [9]. Immunohistochemistry and FISH are first-line methods for determining ALK status [10]. All patients with ALK-positive status should be treated with an ALK inhibitor. Our patient was started on crizotinib. Crizotinib is a first-generation ALK inhibitor. It competes with the adenosine triphosphate and inhibits receptor tyrosine kinases, including ALK, mesenchymal-epithelial growth factor/hepatocyte growth factor receptors, and repressor of silencing 1 [11,12]. Crizotinib poorly crosses the blood-brain barrier and cannot attain adequate levels in the cerebrospinal fluid (CSF) [13]. Visual disorders are the most common side effects of crizotinib [14]. This side effect did occur in our patient. Alternately, she was transitioned to the second-generation ALK inhibitor, alectinib.

Since its introduction into the market, alectinib has become a first-line ALK inhibitor in ALK-positive NSCLC patients, specifically due to its ability to cross the blood-brain barrier and reach a therapeutic concentration in the CSF [15]. In addition, it inhibits ALK phosphorylation and ALK-dependent activation of the downstream pathway, thereby inhibiting the growth of cell lines with aberrant ALK expression [16]. Our patient on alectinib has shown good tolerance, with no reported side effects or metastasis to the brain.

Multiple trials have shown the benefit of crizotinib over chemotherapy. Crizotinib causes an increase in progression-free survival compared to chemotherapy [14,17]. Furthermore, crizotinib is associated with a better quality of life than chemotherapy [17]. However, the overall survival benefit has not been established because many patients cross over to the crizotinib arm in the studies [14,18].

In a meta-analysis that combined the hazard ratio (HR) of progression-free survival from three studies, alectinib was found to have a significantly longer, progression-free survival compared to crizotinib (pooled HR = 0.41; 95% confidence interval [CI] = 0.29-0.53) [19]. Furthermore, the ALEX study has shown the overall survival benefit of alectinib, with a five-year overall survival rate of 62.5% (95% CI = 54.3-70.8) with alectinib and 45.5% (95% CI = 33.6-57.4) with crizotinib [20]. In addition, a pooled analysis of data from two open-label phase II studies (NP28673 [NCT01801111] and NP28761 [NCT01871805]) has reported median overall survival with alectinib to be 29.1 months (95% CI = 21.3-39.0) [21].

Identification of driver mutations and the development of targeted therapies have increased the survival rate of NSCLC. As a result, the two-year relative survival rate of NSCLC increased from 34% during 2009 through 2010 to 42% during 2015 through 2016, with absolute gains of 5-6% for every stage of diagnosis [22]. It has been six years since our patient was diagnosed with advanced NSCLC. She has not had any evidence of active or progressive disease while maintaining a good quality of life.

## Conclusions

ALK inhibitors increase the survival and quality of life in patients with ALK-positive NSCLC. We present an uncommon case of a 37-year-old female six years since her diagnosis of ALK-positive, advanced-stage NSCLC. She was treated with an ALK mutation-directed therapy of crizotinib and, subsequently, transitioned to the second-generation ALK inhibitor, alectinib, due to side effects. This response has been maintained for over six years with a good quality of life.

## References

[1] Sung H, Ferlay J, Siegel RL, Laversanne M, Soerjomataram I, Jemal A, Bray F (2021). Global Cancer Statistics 2020: GLOBOCAN estimates of incidence and mortality worldwide for 36 cancers in 185 countries. CA Cancer J Clin.

[2] (2021). Cancer Stat Facts: lung and bronchus cancer. https://seer.cancer.gov/statfacts/html/lungb.html.

[3] Molina JR, Yang P, Cassivi SD, Schild SE, Adjei AA (2008). Non-small cell lung cancer: epidemiology, risk factors, treatment, and survivorship. Mayo Clin Proc.

[4] (2021). National Cancer Institute: SEER*Explorer application. https://seer.cancer.gov/explorer/application.html.

[5] Jemal A, Miller KD, Ma J (2018). Higher lung cancer incidence in young women than young men in the United States. N Engl J Med.

[6] (2013). A genomics-based classification of human lung tumors. Sci Transl Med.

[7] Soda M, Choi YL, Enomoto M (2007). Identification of the transforming EML4-ALK fusion gene in non-small-cell lung cancer. Nature.

[8] Shaw AT, Yeap BY, Mino-Kenudson M (2009). Clinical features and outcome of patients with non-small-cell lung cancer who harbor EML4-ALK. J Clin Oncol.

[9] Lindeman NI, Cagle PT, Beasley MB (2013). Molecular testing guideline for selection of lung cancer patients for EGFR and ALK tyrosine kinase inhibitors: guideline from the College of American Pathologists, International Association for the Study of Lung Cancer, and Association for Molecular Pathology. J Thorac Oncol.

[10] Lindeman NI, Cagle PT, Aisner DL (2018). Updated molecular testing guideline for the selection of lung cancer patients for treatment with targeted tyrosine kinase inhibitors: guideline from the College of American Pathologists, the International Association for the Study of Lung Cancer, and the Association for Molecular Pathology. Arch Pathol Lab Med.

[11] Heigener DF, Reck M (2018). Crizotinib. Recent Results Cancer Res.

[12] Curran MP (2012). Crizotinib: in locally advanced or metastatic non-small cell lung cancer. Drugs.

[13] Costa DB, Kobayashi S, Pandya SS, Yeo WL, Shen Z, Tan W, Wilner KD (2011). CSF concentration of the anaplastic lymphoma kinase inhibitor crizotinib. J Clin Oncol.

[14] Shaw AT, Kim DW, Nakagawa K (2013). Crizotinib versus chemotherapy in advanced ALK-positive lung cancer. N Engl J Med.

[15] Gadgeel SM, Gandhi L, Riely GJ (2014). Safety and activity of alectinib against systemic disease and brain metastases in patients with crizotinib-resistant ALK-rearranged non-small-cell lung cancer (AF-002JG): results from the dose-finding portion of a phase 1/2 study. Lancet Oncol.

[16] Paik J, Dhillon S (2018). Alectinib: a review in advanced, ALK-positive NSCLC. Drugs.

[17] Solomon BJ, Mok T, Kim DW (2014). First-line crizotinib versus chemotherapy in ALK-positive lung cancer. N Engl J Med.

[18] Solomon BJ, Kim DW, Wu YL (2018). Final overall survival analysis from a study comparing first-line crizotinib versus chemotherapy in ALK-mutation-positive non-small-cell lung cancer. J Clin Oncol.

[19] Yang YL, Xiang ZJ, Yang JH, Wang WJ, Xiang RL (2020). Effect of alectinib versus crizotinib on progression-free survival, central nervous system efficacy and adverse events in ALK-positive non-small cell lung cancer: a systematic review and meta-analysis. Ann Palliat Med.

[20] Mok T, Camidge DR, Gadgeel SM (2020). Updated overall survival and final progression-free survival data for patients with treatment-naive advanced ALK-positive non-small-cell lung cancer in the ALEX study. Ann Oncol.

[21] Ou SI, Gadgeel SM, Barlesi F (2020). Pooled overall survival and safety data from the pivotal phase II studies (NP28673 and NP28761) of alectinib in ALK-positive non-small-cell lung cancer. Lung Cancer.

[22] Siegel RL, Miller KD, Fuchs HE, Jemal A (2021). Cancer statistics, 2021. CA Cancer J Clin.

